# Could successful cryoballoon ablation of paroxysmal atrial fibrillation prevent progressive left atrial remodeling?

**DOI:** 10.1186/1476-7120-10-11

**Published:** 2012-03-19

**Authors:** Tamás Erdei, Mónika Dénes, Attila Kardos, Attila Mihálcz, Csaba Földesi, András Temesvári, Mária Lengyel

**Affiliations:** 1 Semmelweis University, School of Ph.D. Studies, Budapest, Üllői út 26 H-1085, Hungary; 2 Gottsegen Hungarian Institute of Cardiology, Budapest, Haller utca 29 H-1096, Hungary

**Keywords:** Paroxysmal atrial fibrillation, Cryoballoon catheter ablation, Echocardiography, Left atrial remodeling, Left atrial size, Left atrial function

## Abstract

**Background:**

Radiofrequency catheter ablation of atrial fibrillation (AF) has been proved to be effective and to prevent progressive left atrial (LA) remodeling. Cryoballoon catheter ablation (CCA), using a different energy source, was developed to simplify the ablation procedure. Our hypothesis was that successful CCA can also prevent progressive LA remodeling.

**Methods:**

36 patients selected for their first CCA because of nonvalvular paroxysmal AF had echocardiography before and 3, 6 and 12 months after CCA. LA diameters, volumes (LAV) and LA volume index (LAVI) were evaluated. LA function was assessed by: early diastolic velocities of the mitral annulus (Aa_sept_, Aa_lat_), LA filling fraction (LAFF), LA emptying fraction (LAEF) and the systolic fraction of pulmonary venous flow (PVSF). Detailed left ventricular diastolic function assessment was also performed.

**Results:**

Excluding recurrences in the first 3-month blanking period, the clinical success rate was 64%. During one-year of follow-up, recurrent atrial arrhythmia was found in 21 patients (58%). In the recurrent group at 12 months after ablation, minimal LAV (38 ± 19 to 44 ± 20 ml; *p *< 0.05), maximal LAV (73 ± 23 to 81 ± 24 ml; *p *< 0.05), LAVI (35 ± 10 to 39 ± 11 ml/m^2^; *p *= 0.01) and the maximal LA longitudinal diameter (55 ± 5 to 59 ± 6 mm; *p *< 0.01) had all increased. PVSF (58 ± 9 to 50 ± 10%; *p *= 0.01) and LAFF (36 ± 7 to 33 ± 8%; *p *= 0.03) had decreased. In contrast, after successful cryoballoon ablation LA size had not increased and LA function had not declined. In the recurrent group LAEF was significantly lower at baseline and at follow-up visits.

**Conclusions:**

In patients whose paroxysmal atrial fibrillation recurred within one year after cryoballoon catheter ablation left atrial size had increased and left atrial function had declined. In contrast, successful cryoballoon catheter ablation prevented progressive left atrial remodeling.

## Background

The relationship between left atrial (LA) dilatation and atrial fibrillation (AF) has been widely accepted for a long time [1]. Electrophysiological, structural and functional atrial remodeling have been observed in patients with AF, and it has been shown that normal myocardial tissue is often replaced with fibrosis [2]. Dilated left atrium predict later cardiovascular events [3]. Catheter ablation of atrial fibrillation has been established as a therapeutic option for patients with symptomatic, drug-resistant AF [4,5].

Radiofrequency catheter ablation (RFCA) can affect left atrial size and function either from the scar caused by the ablation or from reverse atrial remodeling following conversion to normal sinus rhythm. After successful RFCA of atrial fibrillation, LA size has been found to decrease and LA function and left ventricular diastolic function to improve; the process has been called 'reverse LA remodeling'. In contrast, after an unsuccessful catheter ablation, opposite changes were observed [6-13] and confirmed by a meta-analysis [14].

Cryothermal energy is an alternative energy source that has been developed to overcome some of the disadvantages of RFCA. Cryoenergy offers increased catheter stability, less endothelial disruption with lower thromboembolic risk, and minimal tissue contraction with healing, less generation of inhomogeneous lesions, less oesophageal damage and fewer pulmonary vein stenoses. Balloon-based cryo-technology potentially offers a simpler and faster means of isolating the pulmonary veins that, theoretically, relies less on the dexterity of the operator and causes less scar in the left atrium. Previous studies suggest this is a safe and feasible approach with a similar success rate in patients with paroxysmal AF (PAF) to radiofrequency catheter ablation [15].

Left atrial remodeling has not been examined in the previous studies which used cryoballoon catheter ablation of patients with AF [15,16]. Our hypothesis was that successful cryoballoon catheter ablation of patients with paroxysmal AF similarly to RFCA can also prevent progressive LA remodeling.

## Methods

### Patients

36 consecutive patients with recurrent, symptomatic, nonvalvular PAF were enrolled in the study between June 2008 and July 2009. All had failed to respond antiarrhythmic (propafenone, or amiodarone, or sotalol) or beta blocker therapy. They underwent their first catheter ablation by the cryoballoon technique.

The study was carried out in compliance with the Helsinki Declaration and was approved by the Regional and Institutional Scientific and Research Ethics Committee of the Semmelweis University Budapest (reference number: TUKEB 70/2008). All patients gave written informed consent before participating in the study.

### Cryoballoon ablation

As described in our previous report [17], all patients were treated with a double lumen cryoballoon (Arctic front, Cryocath, Montreal, Quebec), after local anaesthesia and under conscious sedation using boluses of midazolam and fentanyl. At first a decapolar electrophysiological catheter (Bard Electrophysiology Inc., Lowell, MA, USA) was placed in the coronary sinus through the right jugular vein, and a diagnostic quadripolar electrophysiological catheter was introduced through the right femoral vein and positioned in the right ventricle. An intracardiac echocardiography catheter (Acunave, Acuson, Mountain View, CA, USA) was introduced through the left femoral vein and positioned in the right atrium, in order to ensure safe transseptal approach. After the intracardiac echocardiography-guided single posterior transseptal puncture, a circular mapping catheter (Lasso catheter, PV Orbiter, Bard) was advanced and positioned in the antrum of each pulmonary vein to record the presence of pulmonary vein potentials. After registration, the 8 F sheath was exchanged for a 14 F steerable sheath, and the mapping catheter was exchanged for a 28 mm balloon catheter and positioned over an exchange wire to occlude the ostium of each pulmonary vein. At least two 5-minute cryo applications per vein were given to each vein, during using continuous phrenic nerve stimulation when freezing the ostium of the right superior pulmonary vein.

### Study protocol

Antiarrhythmic drugs were stopped 5 half-lives before the ablation, and were not continued afterwards. Oral anticoagulation was before and after ablation, according to the CHADS2-score based on the valid guideline [18].

Comprehensive transthoracic echocardiographic examinations were performed in all patients during sinus rhythm before, and 3, 6 and 12 months after cryoballoon catheter ablation. Transoesophageal echocardiography was performed in all patients before catheter ablation, to exclude left atrial and left atrial appendage thrombus. Transthoracic and transoesophageal echocardiography were performed within 24 hours of each other.

After the ablation, follow-up examinations at 1, 3, 6 and 12 months included clinical history and examination, ECG and 24-hour Holter ECG. A 10-day transtelephonic ECG was performed before the 3-month, 6-month and 12-month follow-up visits.

### Definition of success

Based on the definition of the Expert Consensus Statement on Catheter and Surgical Ablation of Atrial Fibrillation [4], cryoballoon catheter ablation was considered to be clinically successful, if after the initial 3-month blanking period--a time interval during which success is not evaluated--recurrent atrial arrhythmia was not recognized on clinical, or ECG or Holter ECG, or transtelephonic ECG examinations.

In order to evaluate the effect of all episodes of recurrent arrhythmia, recurrences in the blanking period were also taken into account for the final analysis. So the patients were divided into recurrent and arrhythmia-free (AF-free) groups. The recurrent group was defined as patients with recurrent atrial arrhythmia at any time during the follow-up.

### Echocardiography

Transthoracic echocardiography was performed with a General Electric Vivid S6 machine (General Electric, Milwaukee, WI, USA).

Left ventricular (LV) global systolic function was assessed by ejection fraction (EF) evaluated by the modified biplane Simpson's rule. LV longitudinal systolic function was assessed by septal and lateral systolic velocities of the mitral annulus (Sa_sept_, Sa_lat_) using pulsed-wave tissue Doppler echocardiography (TDE).

In order to evaluate LA remodeling LA size and LA function were evaluated before and after ablation. LA volumes were calculated by two-dimensional echocardiography by the biplane area-length method. Maximal LA volume (LAV_max_) was obtained at left ventricular end-systole, just before mitral valve opening. Minimal LA volume (LAV_min_) was determined at left ventricular end-diastole. LA areas (A_1_, A_2_) and superoinferior longitudinal diameters were measured from apical 4- and 2-chamber views. LA volumes were calculated by the following formula [19]: LAV = 8/3π*A_1_*A_2_/L = 0.85*A_1_*A_2_/L, where L is the shorter superoinferior diameter of the LA. LA volume index (LAVI) was calculated by dividing maximal LA volume by the body surface area (calculated by the Dubois formula, using body height and weight).

The evaluation of LA function by echocardiography is not standardized; different methods have been used to assess LA function in research and in clinical practice [20,21]. The left atrium serves multiple functions, acts as a reservoir during left ventricular systole; as a conduit for blood transiting from the pulmonary veins to the LV during early diastole and as an active contractile pump that augments LV ventricular filling in late diastole.

In the present study four TTE methods were used in the assessment of LA function.

LA booster pump (contractile) function was assessed by:

a) the LA filling fraction (LAFF) which is the ratio of the velocity time integral (VTI) of the late diastolic A wave velocity of mitral inflow to the VTI of the early and late diastolic velocities of mitral inflow (LAFF = VTI_A_/VTI_E+A_) and

b) septal and lateral velocities of the mitral annulus during atrial contraction (Aa_sept_, Aa_lat_), measured by pulsed-wave TDE.

LA reservoir function was assessed by:

a) LA total emptying fraction (LAEF = (LAV_max_-LAV_min_)/LAV_max_) and

b) the systolic fraction of pulmonary venous flow (PVSF = VTI_S_/VTI_S+D_). Pulmonary venous flow was measured within right upper pulmonary vein.

More recent methods of evaluating LA function, such as colour-coded tissue-Doppler based strain and strain-rate and two-dimensional speckle-tracking based strain and strain rate were not available in our institute during the study period.

In the detailed evaluation of left ventricular diastolic dysfunction, we used the methods described in recent recommendations [22,23]. Septal and lateral early diastolic velocities of the mitral annulus (Ea_sept_, Ea_lat_) were measured by TDE, which characterize left ventricular relaxation. In the assessment of mean left ventricular filling pressure E/Ea ratios were calculated, using both the lateral, septal and average Ea velocity. Early (E) and late diastolic (A) velocities of mitral inflow, and deceleration time (DT) were measured by pulsed wave Doppler echocardiography, and E/A ratio was calculated. Systolic (S) and diastolic (D) velocities of pulmonary venous flow were measured and S/D ratio was also calculated.

### Statistical analysis

Data are shown as mean ± standard deviation (SD) (95% confidence interval). Unpaired *t*-test was used to compare means of continuous variables between unrelated groups. Chi-square analysis was used to compare patient group characteristics with discrete variables. Paired-sample Student's *t*-test was used to evaluate changes in echocardiographic parameters of LA size and function and of LV diastolic function during the follow-up period within groups. A *p *< 0.05 was considered statistically significant for all calculations. Statistical analysis was performed using SPSS (Statistical Software for Social Sciences) version 13.0.

## Results and discussion

### Procedural outcome

The clinical success rate, as it was defined previously with no recurrence after the 3-month blanking period [4], was 23 out of 36 patients (64%). Repeat ablation had not been performed during the first year follow-up.

15 patients were totally free of arrhythmias throughout the first 12 months of follow-up (the AF-free group). The other 21 patients (the recurrent group) experienced some recurrence of atrial arrhythmias. Only early (within 3 months after treatment) recurrence in 8, only between 3 and 12 months after treatment recurrence in 4, both early and 3 to 12 months recurrences in 9 patients were detected.

### Baseline characteristics

#### Baseline clinical characteristics

36 consecutive patients were enrolled in the study; there were 26 males and the mean age was 57.4 ± 8.9 years. The most frequent symptom was palpitation; signs or symptoms of congestive heart failure were not present. 27 patients (75%) had hypertension. The average time period from the first recognition of AF was 6.7 ± 7.3 years (range 0.5-20).

In patients who later developed a recurrence (recurrent group), had a longer duration of atrial fibrillation (8.8 ± 8.7 vs 3.8 ± 3.3 years, *p *< 0.05) before cryoballoon catheter ablation. At baseline, patients' age, gender, prevalence of concomitant diseases such as hypertension, diabetes mellitus, hyperlipidemia and obesity were not significantly different between the recurrent and AF-free groups (Table 1).

**Table 1:** Baseline clinical and echocardiographic characteristics

	Total study population (n = 36)	Recurrent group (n = 21)	AF-free group (n = 15)	Normal values from the literature	Reference number
***Clinical characteristics ***					

**Age (yrs)**	57.4 ± 8.9	59.6 ± 5.8	54.2 ± 11.5	-	-

**Duration of AF(yrs)**	6.7 ± 7.3	8.8 ± 8.7	3.8 ± 3.3*	-	-

**Male (%)**	26 (72%)	13 (62%)	13 (87%)	-	-

**Hypertension (%)**	27 (75%)	15 (71%)	12 (80%)	-	-

**Obesity (%)**	10 (28%)	3 (14%)	7 (47%)	-	-

**Ischaemic HD**	4 (11%)	3 (14%)	1 (7%)	-	-

***Echocardiographic characteristics ***					

**LV EF (%)**	63 ± 5	64 ± 5	62 ± 6	≥ 55%	[19]

**LAV_max _(ml)**	71 ± 21	73 ± 23	67 ± 20	< 56	[19]

**LAVI (ml/m^2^)**	34 ± 10	35 ± 10	33 ± 9	< 29	[19]

**LAV_min _(ml)**	36 ± 17	38+19	30 ± 12	25 ± 13	[24]

**LA SI diam (mm)**	55 ± 5	55 ± 5	54 ± 6	-	-

**LAEF (%)**	51 ± 10	48 ± 11	55 ± 8*	56 ± 8	[24]

**LAFF (%)**	35 ± 7	36 ± 7	34 ± 7	41 ± 14	[24]

**PVSF (%)**	59 ± 8	58 ± 9	60 ± 7	60 ± 9	[25]

**Aa_lat _(cm/s)**	10.1 ± 2.3	9.7 ± 2	10.7 ± 2.7	-	-

**Aa_sept _(cm/s)**	9.1 ± 2	8.8 ± 1.8	9.7 ± 2.1	-	-

**Ea_lat _(cm/s)**	11.1 ± 2.8	11.2 ± 2.4	10.9 ± 3.2	> 10	[23]

**Ea_sept _(cm/s)**	8.4 ± 2.3	8.1 ± 1.9	8.8 ± 2.8	> 8	[23]

**E/Ea_avg_**	7.3 ± 2	7.2 ± 1.8	7.3 ± 2.1	< 8	[23]

*: *p *< 0.05, recurrent vs AF-free group

Abbreviations:

*Aa_lat_*; late diastolic velocity measured by pulsed-wave tissue Doppler echocardiography at the lateral mitral annulus

*Aa_sept_*; late diastolic velocity measured by pulsed-wave tissue Doppler echocardiography at the septal mitral annulus

*AF*; atrial fibrillation

*E*; early diastolic velocity of mitral pulsed wave Doppler inflow

*Ea_avg_*; average value of septal and lateral early diastolic velocities measured by pulsed-wave tissue Doppler echocardiography

*Ea_lat_*; early diastolic velocity measured by pulsed-wave tissue Doppler echocardiography at the lateral mitral annulus

*Ea_sept_*; early diastolic velocity measured by pulsed-wave tissue Doppler echocardiography at the septal mitral annulus

*EF*; ejection fraction *HD*; heart disease

*LAEF*; left atrial total emptying fraction

*LAFF*; left atrial filling fraction

*LAV*; left atrial volume

*LAVI*; left atrial volume index

*LAV_max_*; left atrial maximal volume

*LAV_min_*; left atrial minimal volume

*LV*; left ventricular

*PVSF*; systolic fraction of pulmonary venous flow

#### Baseline echocardiographic characteristics

Left ventricular EF was 63 ± 5% (55-72%). Left ventricular and left atrial dimensions, and left atrial functional parameters, TDE parameters of LV diastolic dysfunction and normal values from the literature [19,23-25] are shown in Table 1.

Septal and lateral early diastolic velocities of the mitral annulus (Easept, Ealat), E/Ea ratios, LA size and LA pump function were not significantly different between the recurrent and AF-free groups. However, the left atrial reservoir function by left atrial total emptying fraction was significantly lower in the recurrent group (55 ± 8 vs 48 ± 11%, *p *< 0.05) (Table 1).

### Follow-up results

#### Left atrial size

**Figure 1:**
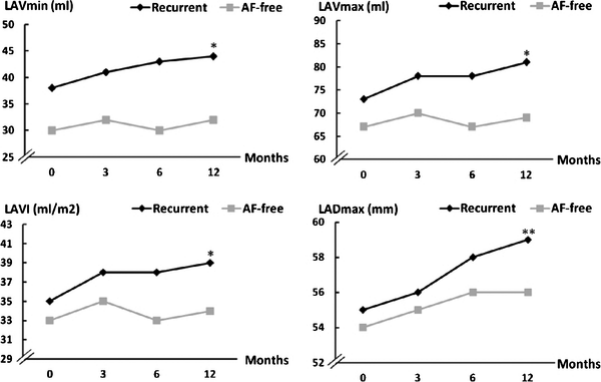
**Changes in left atrial dimensions and volumes after cryoballoon ablation**. (*: *p *< 0.05, baseline to 12 months; **: *p *< 0.01, baseline to 12 months; SD values are in Table 2. LAV_max_: left atrial maximal volume, LAV_min_: left atrial minimal volume, LAVI: left atrial volume index, LADmax: maximal longitudinal superoinferior diameter).

In the recurrent group, at 12 months after ablation, the minimal left atrial volume (38 ± 19 to 44 ± 20 ml; *p *< 0.05), the maximal left atrial volume (73 ± 23 to 81 ± 24 ml; *p *< 0.05), LAVI (35 ± 10 to 39 ± 11 ml/m^2^; *p *= 0.01) and the maximal left atrial longitudinal diameter (55 ± 5 to 59 ± 6 mm; *p *< 0.01) had all increased (Figure 1). In contrast, after successful cryoballoon ablation, LA size had not increased (Table 2).

**Table 2:** Follow-up results

	Recurrent				AF-free			
	
	Baseline	3	6	12	Baseline	3	6	12
**LAV_min _(ml)**	38 ± 19	41 ± 16	43 ± 18	44 ± 20^a^	30 ± 12	32 ± 13	30 ± 11	32 ± 11

**LAV_max_ (ml)**	73 ± 23	78 ± 29	78 ± 23	81 ± 24^a^	67 ± 20	70 ± 20	67 ± 19	69 ± 15

**LAVI (ml/m^2^)**	35 ± 10	38 ± 13	38+10	39 ± 11^a^	33 ± 9	35 ± 8	33 ± 8	34 ± 7

**LAD_max_ (mm)**	55 ± 5	56 ± 6	58 ± 6	59 ± 6^b^	54 ± 6	55 ± 5	56 ± 4	56 ± 5

**LAFF (%)**	36 ± 7	35 ± 8	34 ± 6	33 ± 8^a^	34 ± 7	35 ± 11	38+11	32 ± 8

**Aa_sept _(cm/s)**	8.8 ± 1.8	8.3 ± 2.1	8.6 ± 1.8	8.8 ± 2.2	9.7 ± 2.1	10.3 ± 3.1^c^	10.4 ± 1.7^c^	9.7 ± 2.1

**Aa_lat _(cm/s)**	9.8 ± 2.1	10.1 ± 3.4	10.2 ± 2.7	10.2 ± 2.7	10.7 ± 2.7	10.7 ± 2.2	11.5 ± 2.1	10.8 ± 3.1

**LAEF (%)**	48 ± 11	47 ± 13	45 ± 10	47 ± 11	55 ± 8^c^	55 ± 8^c^	56 ± 8^d^	55 ± 9^c^

**PVSF (%)**	58 ± 9	55 ± 8	55 ± 9	50 ± 10^a^	60 ± 7	56 ± 9	61 ± 9	60 ± 10

**Ea_sept _ (cm/s)**	8.1 ± 1.9	8.8 ± 1.6	8.9 ± 1.9	7.7 ± 1	8.8 ± 2.8	9 ± 2.5	8.6 ± 2.3	8.8 ± 2.3

**Ea_lat _ (cm/s)**	11.2 ± 2.4	12 ± 2.5	12 ± 2.8	11.3 ± 1.6	10.9 ± 3.2	11.6 ± 3.5	11.6 ± 3.6	11.7 ± 3.1

**E/Ea_avg _**	7.2 ± 1.8	7.3 ± 2.	8 ± 2.6	8.5 ± 2.3^b^	7.3 ± 2.1	7.4 ± 2	7 ± 1.5	7.4 ± 2.4

^a^: *p *< 0.05, baseline to 12 months;

^b^: *p *< 0.01, baseline to 12 months;

^c^: *p *< 0.05, recurrent vs AF-free at the same follow-up visit;

^d^: *p *< 0.01, recurrent vs AF-free at the same follow-up visit.

Abbreviations:

*Aal_at_*; late diastolic velocity measured by pulsed-wave tissue Doppler echocardiography at the lateral mitral annulus

*Aa_sept_*; late diastolic velocity measured by pulsed-wave tissue Doppler echocardiography at the septal mitral annulus

*E*; early diastolic velocity of mitral pulsed wave Doppler inflow

*Ea_avg_*; average value of septal and lateral early diastolic velocities measured by pulsed-wave tissue Doppler echocardiography

*Ea_lat_*; early diastolic velocity measured by pulsed-wave tissue Doppler echocardiography at the lateral mitral annulus

*Ea_sept_*; early diastolic velocity measured by pulsed-wave tissue Doppler echocardiography at the septal mitral annulus

*LAD_max_*; maximal left atrial longitudinal diameter

*LAEF*; left atrial total emptying fraction

*LAFF*; left atrial filling fraction

*LAVI*; left atrial volume index

*LAV_max_*; left atrial maximal volume

*LAV_min_*; left atrial minimal volume

*PVSF*; systolic fraction of pulmonary venous flow

#### Left atrial function

**Figure 2:**
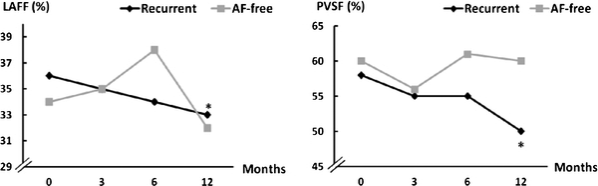
**Changes in left atrial pump and reservoir function after cryoballoon ablation**. (*: p < 0.05, baseline to 12 months; SD values are in Table 2. LAFF: left atrial filling fraction; PVSF: systolic fraction of pulmonary venous flow).

**Figure 3:**
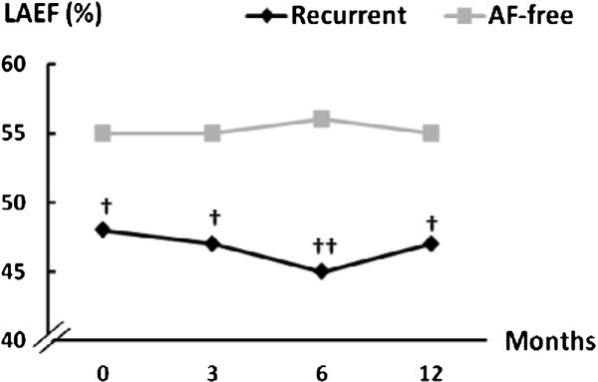
**Left atrial total emptying fraction (LAEF) during the follow-up**. (†: p < 0.05, recurrent vs AF-free at the same follow-up visit; ††: p < 0.01, recurrent vs AF-free at the same follow-up visit; SD values are in Table 2).

In the recurrent group, at 12 months after ablation, the left atrial reservoir function as assessed by the systolic fraction of pulmonary venous flow (58 ± 9 to 50 ± 10%; *p *= 0.01) and LA pump function assessed by left atrial filling fraction (36 ± 7 to 33 ± 8%; *p *= 0.03) had decreased (Figure 2). Left atrial total emptying fraction, which is a parameter of LA reservoir function, did not change significantly during the follow-up; however, it was significantly lower at all follow-up visits in the recurrent group (Figure 3).

In contrast, after successful cryoballoon ablation, LA function had not declined (Table 2).

#### Left ventricular diastolic function

**Figure 4:**
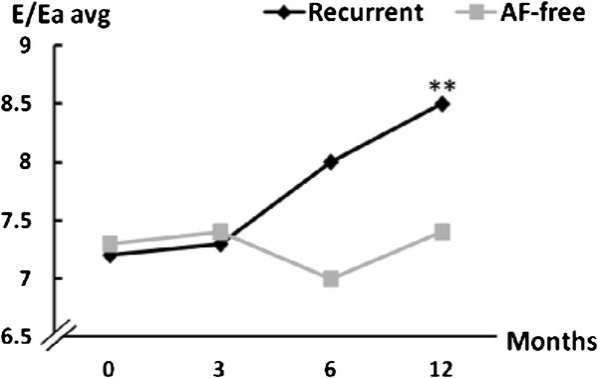
**E/Ea average during the follow-up**. (**: p < 0.01, baseline to 12 months; SD values are in Table 2).

Lateral and septal early diastolic velocities did not change significantly during the follow-up (Table 2). Whereas, E/Eaaverage, which was used to assess the mean LV filling pressure, was increased at one year from baseline values (7.2 ± 1.8 to 8.5 ± 2.3; *p *= 0.005) (Figure 4).

## Discussion

Our study was the first in which left atrial structural and functional remodeling were examined in detail in patients who had cryoballoon catheter ablation for paroxysmal atrial fibrillation. Successful ablation prevented progressive LA remodeling, whereas the left atrium enlarged in patients with recurrent atrial arrhythmias.

### Left atrial size before ablation

In the present study, left atrial size before cryoballoon ablation did not affect the success rate; there was no difference in LA size before ablation between AF- free and recurrent group of patients. These results are similar to a study by Rodrigues et al. [26] in which PAF patients underwent radiofrequency catheter ablation and the LA volume before ablation was not different between the successful and unsuccessful groups. However, in some other studies LA enlargement before treatment, detected by different imaging modalities has been identified as a risk factor for AF recurrence after radiofrequency catheter ablation [13,27-30].

### Left atrial structural remodeling

We found that at one year after unsuccessful cryoballoon catheter ablation left atrial size had increased. In contrast, successful ablation had prevented progressive LA dilatation. The meta-analysis of 17 studies by Jeevanantham et al. [14] confirmed that in patients who had successful radiofrequency catheter ablation of paroxysmal and/or persistent atrial fibrillation LA diameter and volume decreased, which is described as LA structural reverse remodeling. After unsuccessful ablation, LA size did not change or non-significantly increased.

In patients with PAF, LA structural reverse remodeling has not always been observed even after successful catheter ablation. Beukema et al. [8] found that at six months left atrial anteroposterior and superoinferior diameters had decreased in patients who remained in sinus rhythm. Donal et al. [31] investigated 31 lone PAF patients after successful RF ablation, and at 1 year also found a reduced mean left atrial anteroposterior diameter and LA volume index. Reant et al. observed that LA area had decreased in lone PAF patients 11 months after successful RF ablation; but LA area had also decreased after unsuccessful ablation [6]. Rodrigues [26] et al. observed that after radiofrequency catheter ablation of PAF, there was no significant change of the LA maximal volume 8 ± 2 months after ablation, either in the successful, or in the unsuccessfully treated groups. In their study the prevalence of hypertension was 39%. In our study the high prevalence of associated hypertension (80%), which is a well-known determinant of left atrial size, and the limited sample size (AF-free group: N = 15), probably contributed to the lack of reverse remodeling. Another potential explanation could be that the left atrium of the AF-free patients might have been remodeled to such an extent during the years in which they had paroxysmal atrial fibrillation (3.8 ± 3.3 years), that the one-year follow-up period was not long enough for significant reverse remodeling which could be recognised by two-dimensional transthoracic echocardiography, especially in such a small sample (AF-free group: N = 15).

### Left atrial functional remodeling

#### Left atrial booster pump function

In our study the left atrial filling fraction--a parameter of LA pump function--decreased 12 months after ablation in the recurrent group. After successful cryoballoon ablation, LA pump function did not change.

In previous studies, after successful radiofrequency ablation in patients with paroxysmal and/or persistent AF, LA pump functional parameters, such as LA filling fraction [6,11], and TDE derived Aa [32] were found to improve.

In PAF patients who underwent successful radiofrequency catheter ablation, similarly to our study, Aa velocities did not change in a study by Rodrigues et al. 8 ± 2 month after RFCA [26]. In studies by Reant et al. [6,7], neither Aa velocities, nor LAFF changed 12 months after successful RFCA. Whereas, Donal et al. found an increase of Aa velocity one year after successful ablation [31], in addition to an increase in the more sophisticated LA pump functional parameter of end-diastolic strain rate.

#### Left atrial reservoir function

In our study the left atrial total emptying fraction--a variable of left atrial reservoir function--was lower at baseline and during follow-up in the recurrent group, but it did not change in either group.

After 12 months the pulmonary venous systolic fraction--a parameter of LA reservoir function--decreased in the recurrent group. After successful cryoballoon ablation, it did not change. In other studies [6,33] as confirmed by a meta-analysis [14] LA reservoir function, assessed by LA total emptying fraction, declined in patients whose arrhythmia recurred.

In PAF patients who underwent successful radiofrequency catheter ablation, left atrial total emptying fraction did not change significantly after 8 months [26] or one year in another studies [6,7]. However, improving LA compliance was shown by Donal et al. a year after successful ablation by increasing LA lateral strain and systolic strain rate [31].

### Left ventricular diastolic function before and after ablation

Left ventricular diastolic function did not affect the success rate in the present study; no difference was found between the groups regarding LV diastolic function at baseline.

We did not demonstrate any change in Ea velocities during the one-year follow-up in the recurrent, or in the AF-free group, similarly to the study of patients with successful PAF ablation by Donal et al. [31]. However, in the studies by Reant et al. [6,7] Ea was found to increase following successful radiofrequency catheter ablation in PAF patients during a 11-month follow-up.

In our study the E/Eaaverage was increased at one year after cryoballoon catheter ablation in the recurrent group. E/Ea ratio was shown to decrease [6,7] or did not change [31] in other studies after successful radiofrequency catheter ablation in PAF patients

## Limitations

The number of patients in our study was relatively small, similar to other studies of this topic [6,7,26]; but we studied a very homogeneous population of patients with exclusively paroxysmal, nonvalvular atrial fibrillation and with normal left ventricular ejection fraction.

The post-procedure monitoring of AF recurrences was fairly intensive in our present study; but it may have missed asymptomatic recurrences.

We used 2DE to calculate LAV, while measurements with magnetic resonance imaging (MRI) or multislice computerized tomography (CT) are more accurate: all echocardiographic methods systematically underestimate LAV [34,35]. However, acquisition of MRI images is time consuming, and the use of multislice CT requires contrast agents and high radiation exposure. In addition, follow-up studies with MRI or CT would be very costly. 2DE methods are safe, widely available, inexpensive, feasible and reliable both for research purposes and clinical practice, especially during follow-up [36]. During long-term monitoring of left atrial remodelling, echocardiography might be useful to detect the chronic consequences of both symptomatic and silent recurrent atrial arrhythmias.

Several approaches are used to measure LAV by 2DE, which makes the comparison of our data with other studies more challenging. LA volumes measured in our study seemed to be larger than some other studies [37,38], which perhaps influenced the success rate during follow-up which was the bottom of the normal range known from the literature [15].

Usually, 20 to 30% of the patients with clinically unsuccessful ablation undergo a redo procedure. According to the definition of the Ablation Consensus Statement [4] in our study only those 13 patients were clinically unsuccessful who had recurrent arrhythmia after the first 3-month blanking period. In that group during the one-year follow-up period there was no redo ablation, mainly because of the limited availability and costs of the procedure. After the one-year follow-up period, 5 out of the 13 unsuccessful patients (38%) underwent a repeat ablation procedure (by radiofrequency catheter ablation).

## Conclusions

In patients whose nonvalvular paroxysmal atrial fibrillation recurred within one year after cryoballoon catheter ablation, left atrial size increased, left atrial pump and reservoir function declined, and the left ventricular filling pressure increased, all related to left atrial remodeling (progressive enlargement). In contrast, successful cryoballoon catheter ablation prevented progressive left atrial remodeling.

It would have been interesting to compare the effect of cryoballoon catheter ablation and RFCA on the LA remodeling which can be subject of a further study.

## Abbreviations

2DE: Two-dimensional echocardiography; A: Late diastolic velocity of mitral pulsed wave Doppler inflow; A_1_: Left atrial area from apical four-chamber view; A_2_: Left atrial area from apical two-chamber view; Aa_lat_: Late diastolic velocity measured by pulsed-wave tissue Doppler echocardiography at the lateral mitral annulus; Aa_sept_: Late diastolic velocity measured by pulsed-wave tissue Doppler echocardiography at the septal mitral annulus; AF: Atrial fibrillation; CCA: Cryoballoon catheter ablation; CHADS_2_-score: Congestive heart failure; hypertension; age; diabetes mellitus; stroke; CT: Computed tomography; D: Diastolic velocity of pulmonary venous flow; DT: Deceleration time; E: Early diastolic velocity of mitral pulsed wave Doppler inflow; Ea_avg_: Average value of septal and lateral early diastolic velocities measured by pulsed-wave tissue Doppler echocardiography; Ea_lat_: Early diastolic velocity measured by pulsed-wave tissue Doppler echocardiography at the lateral mitral annulus; Ea_sept_: Early diastolic velocity measured by pulsed-wave tissue Doppler echocardiography at the septal mitral annulus; EF: Ejection fraction; LA: Left atrium; LAFF: Left atrial filling fraction; LAEF: Left atrial total emptying fraction; LAV: Left atrial volume; LAV_max_: Left atrial maximal volume; LAV_min_: Left atrial minimal volume; LAVI: Left atrial volume index; LV: Left ventricular; MRI: Magnetic resonance imaging; PAF: Paroxysmal atrial fibrillation; PVSF: Systolic fraction of pulmonary venous flow; RFCA: Radiofrequency catheter ablation; S: Systolic velocity of pulmonary venous flow; SD: Standard deviation; TDE: Tissue Doppler echocardiography; VTI: Velocity time integral.

## Competing interests

The authors declare that they have no competing interests.

## Authors' contributions

ML and TE introduced the study idea. AK, AM, CF made substantial contributions to study conception and design, and performed the ablations. TE acquired the transthoracic echocardiography images, performed the analysis and wrote the manuscript. AT acquired the transoesophageal echocardiography images and reviewed and helped to revise the manuscript. MD helped in the interpretation of the results and statistical analysis. All authors read and approved the final manuscript.
